# Medical Items

**Published:** 1897-02

**Authors:** 


### *MEDICAL ITEMS.*

WANTED.—Copy of the *Confederate Manual of Military Surgery.* Address this JOURNAL, stating price.

THE colored physicians of South Carolina have organized a State medical society.

THE next Pan-American Medical Congress will be held in Caracas, Venezuela, November, 1899.

THE Grady Hospital, Atlanta, has built a children's ward. The Hospital now contemplates a training school for nurses.

THE first number of the American Medico-Surgical Bulletin under the new management, has reached us. It is an improvement on the old. It is now issued as a bi-weekly.

WRITERS ought to be more careful in the selection of the titles of their articles. No one can read everything, hence the title, even if it do not tell the whole tale, at least should not be misleading.—*Pediatrics.*

The next meeting of the American Medical Editors' Association will be held in Philadelphia June 1st and 2d. Dr. Hobart A. Hare, of Philadelphia, is President; Dr. H. Bert Ellis, of Los Angeles, Secretary.

A FRIEND writes: "*The Atlanta Medical and Surgical Journal* is one of my favorites. I became partial to it when it was under the management of Dr. Gray. You are doing good work and making a good journal. I hope you have a host of appreciative readers." Thank, doctor, thanks!

---

DR. H. P. COOPER and Dr. W. S. Elkin are preparing to erect in Atlanta a sanitarium for the reception and treatment of surgical cases. A lot has been secured and plans drawn, and work on the building will begin at an early date. It is expected to be completed in about six months.

---

AT A recent meeting of the Chicago Gynecological Society, the paper of the evening was upon the "Treatment of Hemorrhoids." In the course of the discussion, an eminent member of the society prefaced his remarks with these words: "Gentlemen, the rectum is coming to the front."—*Ex.*

---

THE Fort Wayne (Ind.) *Medical Magazine* and the *Journal of Medical Sciences,* published in the same place, have consolidated their fortunes and destinies, and hereafter will appear as the *Fort Wayne Medical-Journal Magazine.* A very wise step, which might be imitated with advantage in other places.

---

THE *Medical News* says that evidences of degeneracy are beginning to appear even in medical journals. The most recent and mortifying example is presented in the advertisement of a semi-monthly journal which lately offered a hundred-dollar bicycle for the person sending before a certain date the largest number of subscriptions at $2.00 per year.

---

EXPERTS AND EXPERTS.—Client (to lawyer)—I'm afraid the physician's testimony will convict me. Lawyer (reassuringly)—Don't be alarmed about that. I'll read up a little about poison in the stomach, and in ten minutes I'll have the doctor in a cold sweat and make the judge and jury think he is a hired perjurer.—*Druggists' Circular.*

---

NOTICE TO MEMBERS OF THE STATE MEDICAL ASSOCIATION.—You will get a request for a paper for the next meeting. Please decide to write a paper, and go to the Macon meeting, which promises to be one of the best and most pleasant held. You can help make it a success.

---

UNDER the able editorial management of Dr. A. W. Stirling, the ATLANTA MEDICAL AND SURGICAL JOURNAL has gone forward many strides within the past few months. Dr. Stirling, in the capacity of editor *pro tem.* (in the absence of Dr. Grandy), has given eminent satisfaction, and the hundreds of friends of the JOURNAL and its editor congratulate Dr. Stirling upon the splendid way in which he conducted this JOURNAL.—*Atlanta Clinic.*

Which is quite correct and duly appreciated.

---

EVERY now and then we get a letter about like the following: "I never run any bills. I pay cash for everything I get. I did not order your journal this year." This in reply to a dun for the year's journal. A man who will take any journal—not ours alone, for there are others—out of the post-office regularly for eight, nine or ten months, then when asked to pay for it write a letter like the above, has a soul smaller than a mustard seed, and his honesty could not be found in a thousand years' search with a microscope.—*Texas Health Journal.*

---

THE fifth annual meeting of the Tri-State Medical Society of Iowa, Illinois and Missouri, will meet in St. Louis, April 6th, 7th and 8th, 1897. A large number of valuable papers will be read. Dr. Joseph Price, of Philadelphia, will hold the Surgical Clinic, Dr. James T. Whittaker, of Cincinnati, the Medical Clinic, and Dr. Dudley Reynolds, Ophthalmic Clinic. Dr. G. Frank Lydston, of Chicago, will entertain the members with an original story during one of the evening sessions. The officers are: A. H. Cordier, M.D., president; Hugh T. Patrick, M.D., vice-president; G. W. Cale, M.D., secretary, 4403 Washington Boulevard, St. Louis.

---

DEATHS OF GEORGIA PHYSICIANS.—Dr. W. T. Green, of Fort Valley, January 4th. Dr. J. H. Asher, of Atlanta, January 7th. Dr. R. W. Willis, of Madison, January 8th. Dr. Willis served in the Confederate army, under Stonewall Jackson. Dr. S. H. Freeman, near Lawrenceville, January 13th. Dr. Freeman was one of the oldest physicians in the State. He had practiced in Gwinnett and surrounding counties for fifty years.

---

DR. LOUISE D. HOLMES has received her certificate from the Board of Medical Examiners of Georgia giving her the right to practice. She is the first woman physician licensed in Georgia. *Medical Fortnightly.*

This statement is not true, though it has appeared in a number of journals. There were female physicians in Georgia long before this lady came here. Dr. Holmes has left Georgia and has been practicing for several months in St. Louis, the home of the journal above quoted.

---

IT SEEMS that there are certain members of our profession—some of them good men, and in every way, except their newspaper advertising weakness, strong men—who are given to periodical manias for advertising themselves in the daily press. Perhaps some new cure for hydrophobia, an idea obtained during a sojourn in Europe, creeps into their brain and they immediately, through the medium of cigars or a bottle of whisky, creep into the daily newspaper with a long account of a new discovery. A distorted blood cell found under the microscope, and they rush for a reporter.—*Denver Medical Times.*

---

THE annual dinner of the Atlanta Society of Medicine was held at the Aragon hotel on the evening of January 21st, and the occasion was immensely enjoyed by all present. A choice and elaborate menu was served, to which the doctors all did ample justice. Following were the toasts: The Atlanta Society of Medicine, by the president, Dr. G. H. Noble; The General Practitioner, by Dr. L. H. Jones; Specialism in Medicine, by Dr. S. G. C. Pinckney; Medical Journalism, by Dr. L. B. Grandy; The Surgery of To-day, by Dr. H. P. Cooper; Fads—Their Position in the Medical Economy, by Dr. V. O. Hardon; and The Profession of Medicine, by Dr. W. S. Elkin. Appropriate impromptu remarks were made and pleasing anecdotes related also by Drs. Gaston, Hutchins, Baird, Crawford, Duncan, Stirling, Wolff, Champion, Campbell and others. When the doctors dispersed at an early hour the universal feeling was that it was well to have been there.

---

GOLDEN WEDDING OF DR. AND MRS. JOSEPH LECONTE.—On Thursday, January 14, 1847, Dr. Joseph LeConte, of Macon, Ga., was married to Miss Caroline Nesbit, of Milledgeville. After an absence from Georgia for nearly half a century, Dr. and Mrs. LeConte returned to Milledgeville, January 14th, 1897, to celebrate their golden wedding. Joseph LeConte was born in Liberty county, Georgia, 1823. He graduated in the University of Georgia 1841, and studied medicine at the College of Physicians and Surgeons, New York, from which he graduated 1845. He located in Macon, where he practiced medicine three years. He then took up the study of the natural sciences, and in 1852 became Professor of Natural History in the University of Georgia, and later, (1857) he was called to the Chair of Chemistry in the University of South Carolina. Since 1869 he has been Professor of Geology and Natural History in the University of California. The occasion of the golden wedding was attended by a large number of relatives and friends of the aged couple, and handsome presents and telegrams of congratulation were received from many more. We trust the distinguished pair may still see many more anniversaries of their happy wedding.

A SHOWMAN'S STRATEGEM.—In the Cleveland *Plain Dealer* the manager of a traveling opera company tells this story: "I was on the road with a farce comedy company, and we put in two weeks in a certain town. We drew good houses all through the engagement. I don't think we had been there more than one night before the doctor nuisance began. There would come a hurried messenger from the box office to the stage manager with a request that he ask if Dr. Bolus, or whatever his name happened to be, was in the house, and, if he was, to send him to the box office at once. Of course the stage manager couldn't well refuse, and general attention was directed to the medical man, much to his satisfaction. We soon found out that the doctors who were so much in demand were very small medical fry.

"Well, we stood it for a few nights, and then an idea occurred to me. I took a walk up the main street until I came to a certain sign hanging over a stairway. I went up to the office indicated, and had a brief conversation with its inmate, ending it by handing him reserved seat tickets.

"That evening immediately after the first act and before any messenger from the box office had a chance to arrive, I stepped out in front of the curtain and held up my hand. Then in my gravest tones I asked:

"'Is Dr. Chizziold in the house?'

"Immediately a very tall colored man, with a bushy white head and huge silver mounted spectacles, arose in the audience and said:

"'Heah I is, sah.'

"The audience tittered, but I kept my gravity.

"'You are wanted at the box office at once, doctor, in a case which requires your immediate professional attention.'

"As the aged darky ducked to me and hobbled from the room the audience broke into a wild roar.

"Perhaps you will understand the cause of their merriment when I add that the old man was a corn doctor and probably the best known eccentric character in town. There were no more doctors called from that stage during our engagement."—*Factotum.*

